# Public health round-up

**DOI:** 10.2471/BLT.26.010426

**Published:** 2026-04-01

**Conflict deepens health crisis**

The World Health Organization (WHO) warned that escalating conflict is severely straining health systems across the Eastern Mediterranean Region. The Islamic Republic of Iran and Lebanon report rising deaths and thousands of injuries, while attacks on health facilities have killed health workers and reduced access to care. Massive displacement in both countries is creating overcrowded, unsanitary conditions that heighten disease risks. Environmental hazards, service shutdowns and restricted movement of medical supplies are worsening the crisis. With humanitarian needs already extreme and funding insufficient, WHO urges all parties to protect health care, allow humanitarian access and work toward de‑escalation.

**Figure:**
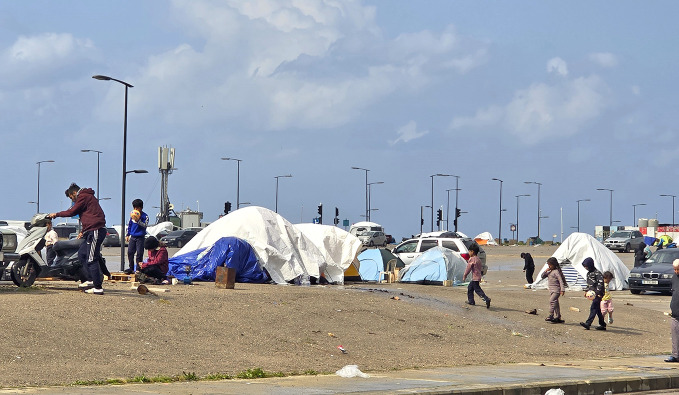
WHO

## Trends in child mortality

A new United Nations report warns that global progress in reducing child mortality is slowing, with an estimated 4.9 million children dying before 5 years of age in 2024. While under‑5 deaths have fallen by more than half since 2000, the rate of decline has dropped by over 60% since 2015. The report, *Levels and trends in child mortality*, offers the most comprehensive assessment to date, including full estimates of causes of death.

Severe acute malnutrition directly caused more than 100 000 deaths among children aged 1–59 months, though the true toll is likely far higher due to underreporting and the indirect effects of weakened immunity. Newborns remain particularly vulnerable, accounting for nearly half of all deaths of children under 5 years of age, largely due to preterm birth, complications during labour and delivery, and infections. 

Infectious diseases, such as malaria, diarrhoea, pneumonia and injuries remain leading causes of death among younger children, while risks shift in adolescence: self-harm is the leading cause of death among girls aged 15–19 years, while road traffic injuries are the leading cause among adolescent boys.

Fragile and conflict‑affected settings face the highest risks, with children nearly three times more likely to die before 5 years of age than those elsewhere. The report urges renewed political commitment, stronger primary health care and sustained financing to reverse the slowdown in progress. 

“History has shown what is possible when the world commits to protecting its children. With sustained investment and political will, we can continue to build on those achievements for future generations,” said Catherine Russell, executive director of the United Nations Children's Fund.

https://bit.ly/4sjL40M

## Addressing drug-resistant bacteria

WHO has released three new target product profiles (TPPs) to guide the development of urgently needed antibiotics targeting some of the world’s most dangerous drug‑resistant infections. The profiles outline the minimum and preferred characteristics for new antibacterial agents aimed at three major priorities: severe multidrug‑resistant Gram‑negative infections, life‑threatening Gram‑positive infections and bacterial meningitis.

Developed through extensive global consultation, the TPPs are intended to help researchers, developers, regulators and funders align innovation with unmet clinical needs. Despite 90 antibacterial agents currently in development, WHO warns that too few target priority pathogens are covered and even fewer medicines represent true innovation. The new guidance aims to close this gap by detailing expectations for efficacy, safety, pharmacokinetics and suitability for vulnerable populations, including newborns, children and immunosuppressed patients.

Drug‑resistant infections such as carbapenem‑resistant *Enterobacterales*, *Acinetobacter baumannii* and *Pseudomonas aeruginosa* continue to drive high mortality and strain health systems. Bacterial meningitis also remains a major global threat, killing one in six affected individuals and leaving many survivors with long‑term disabilities.

The initiative is part of WHO’s partnership with the European Commission’s Health Emergency Preparedness Authority to strengthen the antibiotic pipeline, promote stewardship and ensure equitable access to future treatments.

https://bit.ly/4biG2vj

## Handbook to accelerate hepatitis elimination 

The* Consolidated guidance and implementation handbook on hepatitis B and C *has been published by WHO, offering countries a practical roadmap to expand prevention, testing, treatment and monitoring through a comprehensive public health approach towards elimination of hepatitis B and C. The handbook compiles more than a decade of evidence‑based recommendations into a single operational resource for programme managers, policy-makers, clinicians and partners. It also supports the integration of hepatitis services into primary health care and universal health coverage platforms.

Viral hepatitis remains a major global health threat, with an estimated 254 million people living with hepatitis B and 50 million with hepatitis C. In 2022, hepatitis‑related cirrhosis and liver cancer caused 1.3 million deaths, making hepatitis B and C among the world’s deadliest infectious diseases, despite the availability of highly effective tools, including curative treatment for hepatitis C and vaccines and treatment for hepatitis B.

The new handbook aims to close the gap between available tools and real‑world implementation by providing clear pathways to expand equitable, person‑centred services and strengthen national responses.

“With this first-of-its-kind handbook, WHO is supporting countries to move from evidence-based recommendations to concrete action, reducing new hepatitis infections and combating rising mortality,” said Tereza Kasaeva, director of WHO’s department for HIV, tuberculosis, hepatitis and sexually transmitted infections. 

https://bit.ly/4sY9tsK

## New oral health care guideline

WHO has published a new global guideline to help countries shift toward environmentally friendly, less invasive and more affordable approaches to preventing and managing oral health care. The guidance outlines evidence‑based, mercury‑free clinical interventions that prioritize prevention, non‑invasive care and minimally invasive treatment, while promoting patient safety, quality of care and environmental protection.

Dental caries remains the world’s most common noncommunicable disease, affecting an estimated 2.7 billion people and disproportionately impacting underserved communities. For decades, treatment has relied heavily on dental amalgam, a mercury‑containing material that poses risks to both human health and the environment. The new guideline supports global commitments to phase out mercury under the Minamata Convention and aligns with broader oral health agendas such as the Bangkok Declaration.

The recommendations form a key contribution to the WHO *Global strategy and action plan on oral health 2023–2030*, which calls for integrating essential oral health services into primary health care as part of universal health coverage. By emphasizing prevention, scaling up non‑invasive and minimally invasive care, and promoting safe, affordable alternatives to amalgam, the guideline provides a technical foundation for countries to expand equitable oral health services.

It also aligns oral health with climate and sustainability goals by encouraging environmentally responsible clinical practices.

“This guideline represents a landmark in global oral health,” said Benoit Varenne, WHO dental officer. “For the first time, countries have strong evidence showing that safe and less invasive interventions with mercury-free products can effectively prevent, stop and manage dental caries, while providing a more environmentally sustainable alternative to dental amalgam.” 

https://bit.ly/4bhOPxB

## Health inequality data 

Updated global tools for monitoring health inequalities have been released to help countries better track and address inequities across populations. WHO’s health inequality data repository (HIDR), the world’s largest public collection of disaggregated health inequality data, now includes more than 13 million data points covering over 2 400 health indicators and 22 dimensions of inequality. The update incorporates new data from 62 major international sources, including WHO, Demographic and Health Survey Programme, Eurostat, Global Data Lab, the Institute for Health Metrics and Evaluation, the Organisation for Economic Co-operation and Development, the United Nations Development Programme and the World Bank. 

Alongside the repository update, Version 7 of the health equity assessment toolkit (HEAT and HEAT Plus) introduces enhanced analytical features to support the exploration and reporting of health inequalities. The new “determinants” component allows users to generate scatterplots linking health indicators with social determinants across countries, based on six domains defined in WHO’s framework for monitoring social determinants of health equity: economic security, education, physical environment, social context, health behaviours and health care.

“Providing easy access to the latest global inequality data through the repository and maintaining tools like HEAT to promote the use of these data, are important components of our work on health inequality monitoring,” said Ahmad Reza Hosseinpoor, team lead for health inequality monitoring at WHO. 

Together, the updated HIDR and HEAT tools aim to strengthen national capacity to identify where inequalities exist and inform policies that advance health equity. By improving access to high‑quality disaggregated data and user‑friendly analytical tools, the updates support countries in aligning with WHO’s broader commitment to equity.

https://bit.ly/4smFZ7V

**Cover photo**

A pharmacist collects and prepares medicines from the shelves according to the prescription to support safe and responsible dispensing, Phnom Penh, Cambodia.

**Figure:**
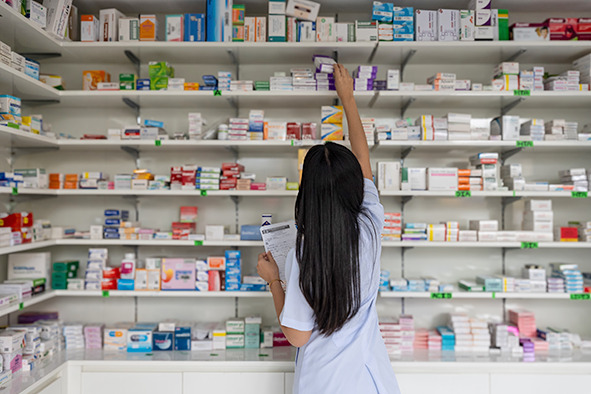
WHO/Enric Catala Contreras

**Looking ahead**

5–7 April. One Health Summit. Lyon, France. https://bit.ly/4bvTJ9b

7 April. World Health Day 2026: together for health, stand with science. Global events. https://bit.ly/4uD1Hps

7–9 April. Global Forum of WHO Collaborating Centres: collaborating for a healthier future. Lyon, France. https://bit.ly/4dsjEAX

